# Time Series Analysis of Cholera in Matlab, Bangladesh, during 1988-2001

**DOI:** 10.3329/jhpn.v31i1.14744

**Published:** 2013-03

**Authors:** Mohammad Ali, Deok Ryun Kim, Mohammad Yunus, Michael Emch

**Affiliations:** ^1^International Vaccine Institute, SNU Research Park, San 4-8 Nakseongdae-dong, Gwanak-gu, Seoul, Korea;; ^2^icddr,b, GPO Box 128, Dhaka 1000, Bangladesh;; ^3^Department of Geography, University of North Carolina-Chapel Hill, Chapel Hill, NC 27599, USA

**Keywords:** Cholera, Climate change, Time series analysis, Matlab, Bangladesh

## Abstract

The study examined the impact of *in-situ* climatic and marine environmental variability on cholera incidence in an endemic area of Bangladesh and developed a forecasting model for understanding the magnitude of incidence. Diarrhoea surveillance data collected between 1988 and 2001were obtained from a field research site in Matlab, Bangladesh. Cholera cases were defined as *Vibrio cholerae* O1 isolated from faecal specimens of patients who sought care at treatment centres serving the Matlab population. Cholera incidence for 168 months was correlated with remotely-sensed sea-surface temperature (SST) and *in-situ* environmental data, including rainfall and ambient temperature. A seasonal autoregressive integrated moving average (SARIMA) model was used for determining the impact of climatic and environmental variability on cholera incidence and evaluating the ability of the model to forecast the magnitude of cholera. There were 4,157 cholera cases during the study period, with an average of 1.4 cases per 1,000 people. Since monthly cholera cases varied significantly by month, it was necessary to stabilize the variance of cholera incidence by computing the natural logarithm to conduct the analysis. The SARIMA model shows temporal clustering of cholera at one- and 12-month lags. There was a 6% increase in cholera incidence with a minimum temperature increase of one degree celsius in the current month. For increase of SST by one degree celsius, there was a 25% increase in the cholera incidence at currrent month and 18% increase in the cholera incidence at two months. Rainfall did not influenc to cause variation in cholera incidence during the study period. The model forecast the fluctuation of cholera incidence in Matlab reasonably well (Root mean square error, RMSE: 0.108). Thus, the ambient and sea-surface temperature-based model could be used in forecasting cholera outbreaks in Matlab.

## INTRODUCTION

Cholera is an acute intestinal disease caused by the bacterium *Vibrio cholerae*. Although knowledge about the epidemiology and ecology of cholera has increased during the last two decades, cholera remains a serious problem in many areas of the world. Cholera appears to be influenced by climatic changes (1-3). In some endemic areas, cholera outbreaks have predictable seasonal patterns. Until recently, the reservoirs or sites for survival and multiplication of *V. cholerae* during inter-epidemic periods were unknown. Recent studies have provided more satisfactory explanations on how seasonality and endemicity of cholera are maintained, providing clues about inter-annual variability (4). Lobitz and others hypothesized that rise in the local sea-surface temperature influences the growth of phytoplankton concentrations, and an increase in sea-surface height increases human-*Vibrio* contact by transporting the bacteria into inland waters through tidal intrusion of plankton (5).

Lobitz *et al.* (5) linked remotely-sensed marine environmental data with cholera incidence in Bangladesh and found that sea-surface temperature (SST) was positively associated with cholera cases. They hypothesized that an increase of SST results in replication of phytoplankton populations, which are directly associated with the increase in *V. cholerae* bacteria and are linked spatially and temporally to zooplankton populations. Indirect measurement of ocean chlorophyll concentration (OCC) is also possible with remotely-sensed satellite imagery (6). However, measurement of the OCC is difficult and possibly problematic for predicting variability across small areas. Locally-measured variables, such as temperature and rainfall, were found to be positively associated with an increase in the number of cholera cases (1,7). Understanding the environmental drivers of cholera outbreaks could facilitate some degree of outbreak prediction, allowing governments to prepare and respond to potential outbreaks (e.g. by employing vaccines). It can also give insight into the local aetiology of cholera and, therefore, help with the planning of prevention strategies.

Huq *et al.* (8) found significant correlations of water temperature, water depth, rainfall, conductivity, and copepod counts with the occurrence of cholera from the data on four rural areas of Bangladesh. However, the environmental drivers for the occurrence of cholera were not same in all those four areas. The lag periods between increases or decreases in temperature and salinity and occurrence of cholera correlate with biological parameters, e.g. plankton population blooms. Hashizume *et al*. (9) found that the number of cholera cases increased with both high and low rainfall in the weeks preceding hospital visits in Dhaka, Bangladesh. Lower temperature predicted a lower incidence of cholera in the first 15 weeks of the year, and low rainfall predicted a peak in spring, and high rainfall predicted a peak at the end of the monsoon (10). However, the mechanisms of this seasonality of cholera are still not fully understood, despite long-standing recognition of the bimodal seasonality in Bangladesh. Hashizume and his colleagues (11) observed an exception­ally-high SST and sea-surface height (SSH), preceding a sharp increase in the number of cholera patients in Dhaka in 1998, suggesting that SST should be taken into account when building predictive models for cholera, using ocean-climate data. Although attempts to predict the incidence of cholera, using ocean-climate data from preceding months, have been made (12-13), an accurate climate-based prediction of cholera epidemics with a longer lag time has not yet been developed. Because of the serious global consequences of cholera and its sensitivity to climate, the World Health Organization has proposed developing an early warning system for cholera epidemics, using climatic parameters (14).

In an earlier study (15) conducted in Bangladesh, ocean chlorophyll concentration was found to be positively and significantly associated with both high cholera outbreaks (more than 70% of outbreaks) and extreme cholera outbreaks (more than 85% of outbreaks); the two-month lag effects were also significantly and positively associated with increase in the magnitude of cholera. SST, rainfall, SSH, and temperature were not significantly associated with magnitude of cholera. The study employed a regression model, using two-month lag-dependent variables, assuming that the environmental factors will have a two-month delayed effect on the outcome. Using time lag without filtering of autocorrelation in time series data may not capture true time dependency between cholera outbreaks and environmental factors, and the results may mislead the reality. Studies of statistical methods have noted that seasonal autoregressive integrated moving average (SARIMA) is an appropriate method for time series data due to its integrated functions for controlling seasonal variation, autocorrelation, and long-term trends (16,17). It has several advantages, in particular, its forecasting capability and richer information on time-related changes (18,19). SARIMA model is also useful for interpreting and applying surveillance data in disease control and prevention (20,21).

In this study, we used the SARIMA time series model to evaluate climate variability and the fluctuation of cholera incidences in Matlab, Bangladesh. The study measured the spatio-temporal association between cholera incidence and satellite-derived SST data, and the association between cholera incidence and *in-situ* data for rainfall and temperature.

## MATERIALS AND METHODS

### Study area

The study site Matlab is located in south-central Bangladesh, approximately 50 km southeast of the capital city Dhaka and is adjacent to where the river Ganges meets the river Meghna, forming the lower Meghna. This is a field research site of the International Centre for Diarrhoeal Disease Research, Bangladesh (icddr,b). The river Dhonagoda flows from north to south, bisecting the study area into two approximately equal parts. Numerous canals also exist in the study area. These canals remain dry in the winter and become full of water during the monsoon. An embankment was built alongside the Dhonagoda and the Meghna, which was commissioned in full at the end of 1989. The embankment was built primarily to protect the area from monsoon flooding so that agricultural activities can be carried out throughout the year. The embankment protects 31% of approximately 210,000 people living in the study area from flooding.

The study area has a tropical monsoon-type climate, with a hot and rainy summer and a dry winter. For practical purposes of this study, three seasons were distinguished in Bangladesh: summer (March-June), rainy (July-October), and winter (November-February). January is the coolest month with temperatures averaging near 26 ^o^C (78 ^o^F), and April is the warmest with temperatures ranging from 33 ^o^C to 36 ^o^C (91 ^o^F to 96 ^o^F). The climate is among the wettest in the world. The study area receives more than 2,100 mm of rain a year. Most of the rain occurs during the monsoon (June-September), and there is little rain in the winter (November-February).

### Population data

The population database of icddr,b in Matlab is the most comprehensive longitudinal demographic database on a large population in the developing world (15). A health and demographic surveillance system (HDSS) has recorded all vital events of the area's population since 1966. The population in the study area was approximately 210,000 during the study period.

### Cholera data

**Figure 1.**
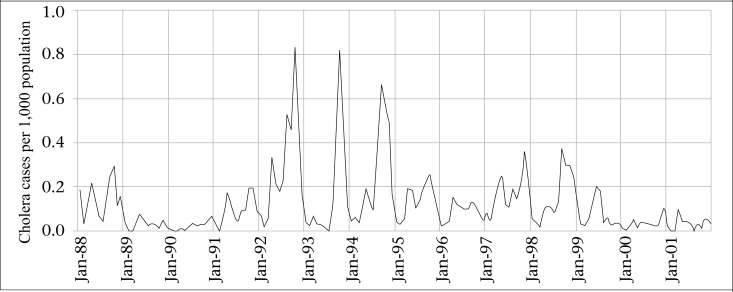
Temporal patterns of cholera incidence, Matlab, 1988-2001

Diarrhoea surveillance data were obtained for all individuals living in the Matlab study area from 1988 to 2001. Cholera cases were defined as *Vibrio cholerae* isolated from faecal specimens of patients who sought care at treatment centres serving the Matlab population. Cholera cases were aggregated by month. The cholera incidence in a month was calculated as the number of cases in the month divided by the mid-year population of the study area in the year, and the rates are expressed as cases per 1,000 population. Figure 1 shows the fluctuations of monthly cholera incidence during the study period.

### Environmental data

We obtained AVHRR (Advanced Very High Resolution Radiometer) satellite-derived SST data for 1985-2001 from NASA's Jet Propulsion Laboratory. The satellite sensor collects SST data at 4 km area. *In-situ* environmental data include monthly ambient temperature and rainfall that were made available for the study period (1985-2001) from a weather station in Chandpur, which is located just outside the study area. We specifically selected these three environmental variables for this study because these variables are often found to be predictors of the magnitude of cholera in various parts of the world (5,7,15,22).

### Analytical methods

We analyzed the data by month, incorporating 168 time-points during the study period (1988-2001). The data prior to 1988 were avoided because there was an oral cholera vaccine trial in early 1985 that offered direct (23) and indirect protection (24) to people of the area. A SARIMA model was used for evaluating the effects of environmental variables on cholera transmission and assessing the ability of the model for forecasting cholera trasmission in the study area. Using data from 1988 to 2000, we fitted the model to cholera incidence (cases per 1,000 people), and then the fitted model was used in predicting cholera cases for the year 2001.

We used the Box-Jenkins modelling strategy (25) to conduct a time series analysis. First, we evaluated the need for variance-stabilizing transformation by simple inspection of the graph of the untransformed series and practical tool, which is the mean-range plot (the range is plotted against the means for each seasonal period) of the untransformed and some transformed series (e.g. logarithm or square root). If the mean-range plots display a random scatter around a straight line, the logarithm transformation is needed (26). We determined the order of non-seasonal (p,d,q) and seasonal (P,D,Q) autoregressive (AR) parameters (p and P) and moving average (MA) parameters (q and Q) as well as the need for non-seasonal and seasonal differencing (d and D) and seasonal period (s), using the following tools: (i) plot of cholera incidence and unit root test, which assists with the non-seasonal and seasonal differencing; (ii) autocorrelation (ACF) and partial autocorrelation (PACF) functions, which indicate the temporal dependence structure in the stationary time series; (iii) Akaike Information Criterion (AIC), which assists in the goodness-of-fit of the model penalizing for the number of parameters; (iv) Ljung-Box test, which measures the ACF of the residuals; and (v) significance of the parameters.

We estimated the parameters of the SARIMA model by maximum likelihood.

The goodness-of-fit of the model was determined for appropriate modelling, using both time series (ACF and PACF of residuals) and classic tools, which checked for the normality of the residuals (27). Finally, we graphically compared the model's fitted values with the observed data to check if it, indeed, models the cholera incidence. We assessed the predictive power of forecasting ability of the method, using the root mean square error (RMSE) criterion.

## RESULTS

**Figure 2.**
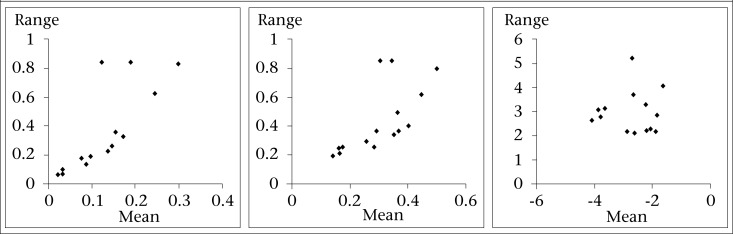
Mean-range plot for cholera incidence per 1,000 population for non-transformed data (left), square-root transformation data (middle), and log-transformed data (right), Matlab, 1988-2001

**Figure 3.**
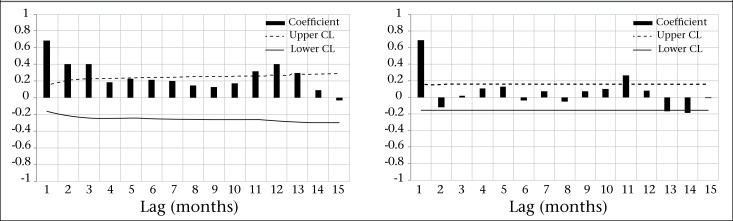
Autocorrelation function (left) and partial autocorrelation (right) of log-transformed cholera incidence in Matlab, 1988-2001

There were 4,157 cholera cases during the study period, with an average of 1.4 cases per 1,000 people. The characteristics of the data are shown in Table 1. The time series of cholera incidence shows that there was no trend in cholera rates during the study period (Figure 1). Through the mean-range plots for each seasonal period (12 months), we found it necessary to stabilize the variance of cholera incidence by computing its natural logarithm (Figure 2). All further statistical procedures, descriptive and analytical, were performed on the logarithmically-transformed cholera incidence. The plots of the sample ACF describes temporal dependence of cholera incidence at lag 1, 2, and at lag 11, 12, 13, and PACF describes the temporal dependence of cholera incidence at lag 1 and at lag 11 (Figure 3), suggesting that non-seasonal and seasonal parameters are needed in the model to account for the temporal autocorrelation in the data. Upon using the Augmented Dickey-Fuller Unit Root Test and checking ACF and PACF (28,29), we found that the data needed one-month non-seasonal differencing and 12-month seasonal differencing to make the data stationarity. Finally, we fitted the SARIMA (0,1,2) (0,1,1)_12_ model, which was found to be the best for these data.

**T1able 1.** Characteristics of the study data, Matlab, 1988-2001

Variable (monthly average)	No. of months	Mean	SD	Minimum	Maximum
Monthly cholera cases (number)	168	24.74	30.53	0	175
Population (number)	168	209,620	6,330	199,511	219,873
Minimum temperature (in degree celsius)	168	20.98	4.79	10.30	27.30
Maximum temperature (in degree celsius)	168	30.21	2.89	22.40	35.10
Rainfall (in mm)	168	168.65	156.36	0	746.0
Sea-surface temperature (in degree celsius)	168	28.34	1.93	24.3	31.95
SD=Standard deviation					

Inter-correlation among the independent variables, using a 12-month seasonal differencing, shows that minimum and maximum temperatures were significantly correlated with each other (p<0.01; Table 2). Maximum temperature was significantly and negatively correlated with rainfall (p<0.01). The results of the cross-correlations, using 12-month seasonal differencing, show that cholera incidence was positively associated (p<0.01) with minimum temperature in the current month, negatively associated (p<.05) at a lag of three months, and positively associated (p<0.05) at a lag of five months (Table 3). Maximum temperature was positively associated (p<0.05) with cholera incidence in the current month, negatively associated (p<0.05) at a lag of one-month, positively associated (p<0.05) at a lag of two months, and again negatively associated (p<0.05) at a lag of three months. SST was negatively associated (p<0.05) with magnitude of cholera at a lag of two months (Table 3). The data on average maximum temperature did not fit well in our model as seen in a study (30); therefore, we used the average minimum temperature in our model. The results of the SARIMA model showed that minimum temperature during the current month and SST at current month and at 2-month lag significantly influenced (p<0.01) cholera incidence (Table 4).

**Figure 4.**
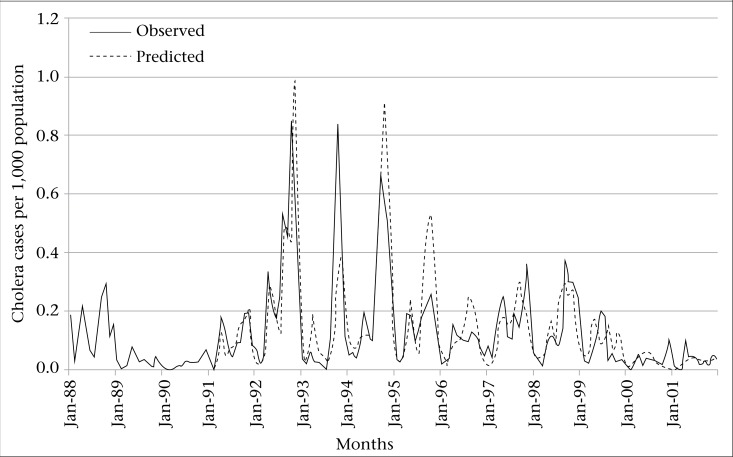
Predicted and observed monthly cholera incidence rate, Matlab,1989-2001

**Figure 5.**
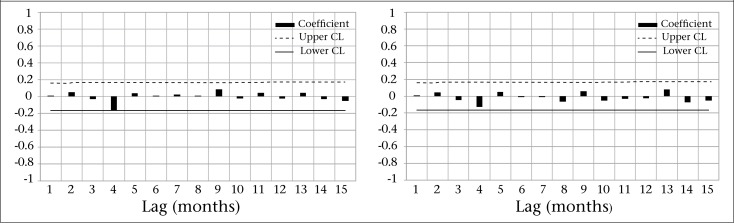
ACF (left) and PACF (right) of residuals from the SARIMA model

By using the SARIMA model with only minimum temperature variable in the equation, it showed that, with a minimum temperature increase of one degree celsius in the current month, there was a 6% increase in cholera incidence [exp (b)=1.06, p<0.01, caeteris paribus]. For increase of SST by one degree celsius, there was a 25% increase in the cholera incidence at currrent month and 18% increase of the cholera incidence at two months [caeteris paribus]. This model was used in predicting cholera incidence from 1989 to 2001, and the results are shown in Figure 4. Since we used moving average from the 12-month seasonal differencing in the SARIMA model, the model eventualy accounts for the data from 1989. It is apparent that the predicted incidence rates were reasonably well-matched with the observed incidence rate. The RMSE, a measure of the size of residuals from the model that used minimum temperature to predict cholera incidence, was 0.108, which means that if the minimum temperature is used in predicting cholera incidence, roughly two-thirds of the residuals will fall between −0.108 and +0.108. The goodness-of-fit analyses show that there was no significant autocorrelation between residuals at different lags in the SARIMA model (Figure 5). The Shapiro-Wilk test for normality also showed that the residuals were distributed normally (p=0.78).

**T1able 2.** Inter-correlations among environmental variables, using 12-month seasonal differencing data, Matlab, 1988-2001

Variable	Maximum temperature	Rainfall	Sea-surface temperature
Minimum temperature	0.31**	-0.13	0.11
Maximum temperature	-	-0.33**	0.07
Rainfall	-	-	-0.11

**p<0.01

**T1able 3.** Cross-correlation coefficients of logarithmic transformation of observed cholera incidence and environmental variables, using 12-month seasonal differencing data, Matlab, 1988-2001

Variable	Lag (month)					
	0	1	2	3	4	5
Minimum temperature	0.26**	-0.01	0.04	-0.15*	-0.01	0.16*
Maximum temperature	0.16*	-0.19*	0.17*	-0.15*	0.02	0.06
Rainfall	-0.1	-0.04	0.04	-0.01	0.05	0.11
Sea-surface temperature	0.12	0.10	-0.15*	-0.07	0.04	-0.01

*p<0.05

**p<0.01

**T1able 4.** Regression coefficients of the SARIMA on the monthly cholera incidence, Matlab, 1988-2001

Parameter	Estimate (b)	Standard error	p value
One-month moving average of cholera incidence (log-transformed)	0.406	0.079	<0.0001
Two-month moving average of cholera incidence (log-transformed)	0.207	0.078	0.008
Seasonal moving average (12-month) of cholera incidence (log-transformed)	0.691	0.081	<0.0001
Minimum temperature at current month	0.062	0.026	0.0015
SST at current month	0.229	0.061	<0.0001
SST at 2-month lag	0.161	0.061	0.008
Akaike Information Criterion (AIC)		362.74	

## DISCUSSION

The results of this study illustrate that fluctuation in the incidence of cholera in Matlab is temperature-driven. The relationship between both ambient and sea-surface temperature and amplification of cholera incidence is well-documented (5-6,8, 30-33). This association likely exists because higher temperatures facilitate *Vibrio* reproduction, which increases concentrations in the aquatic habitat. At greater concentrations, there is an increased probability that people living in the area ingest an infectious dose (6). Previous studies (15,33) conducted in Matlab showed that increased cholera caseload is associated with higher temperature, lower river discharge, and higher ocean chlorophyll concentration. We could not use the data on river discharge and ocean cholera concentration due to lack of reliable datasets. However, as found in a study conducted in Dhaka (9), rainfall has not been found to be an influencing factor for the outbreak of cholera in Matlab, suggesting that the causal pathway of cholera between Dhaka and Matlab is not the same. The link between cholera outbreaks and higher temperatures is an important finding in light of the global warming phenomenon. Climatologists predict a 1.4 °C to 5.8 °C rise in mean temperatures over the next 100 years (34). Increased sea temperature and levels associated with global warming suggest the possibility of increased cholera incidence in many resource-poor regions of the world.

The SARIMA model, based on temperature, forecast cholera incidence in Matlab reasonably well. We used the SARIMA model which is appropriate for analyzing time series data (17); it determines whether AR or MA terms are needed to correct for autocorrelation that remains in the seasonally-differenced data. We found some degree of temporal autocorrelation in the data, which was removed from the stationarized series by adding autoregressive terms (lags of the stationarized series) to the model. The findings of this study, however, lead the authors to believe that seasonal variation in temperature may contribute to cholera incidence in complex ways, presumably through interaction with some unmeasured environmental or behavioural factors (10).

The results of our study show three peaks in the cholera incidence in a month of the year 1993, 1994, and 1995. There was an emergence of new strain of cholera—*V. cholera* O139—in 1993 (35), which co-existed with El Tor for some time and showed a peak in 1993. There are fluctuations of cholera cases by year as well as variations in the seasonal peak in Matlab (36). The three peaks are just the results of the outbreak of *V. cholera* O139 and variations of cholera cases by year and by season. Cholera incidence in the study area is usually higher before and after the monsoon (36,37); thus, we usually see two peaks in a year. This study also found that the lag relationship with the outcome is not linear and one-directional. It is possible that the cholera caseload can be higher at the month of increase in temperature and then can be lower at three months after increase in temperaure in a country, like Bangaldesh.

### Limitations

The limitation of this study is that we could not collect ocean chlorophyll concentration (OCC) for the study period, which is found to be associated with the magnitude of cholera in Bangladesh (16). Also, we could not collect the data on sunshine hour, which is found to be associated with the magnitude of cholera (33). Additionally, since our forecasting model is based on the symptomatic cases and the immunity derived from the mild infections wanes very rapidly, the inapparent infections in the study area may change the patterns of forecasting the disease outbreaks (38).

### Conclusions

Albeit with the limited datasets, this paper supports the premise that the magnitude of cholera in Matlab is temperature-driven, both ambient and sea-surface temperature. A perfect model for predicting cholera outbreaks in the area based on only environmental variables is difficult because the disease may have complex relationships with local population dynamics, the population's health behaviour, and health practices. The results of this study may provide a basis for predicting cholera epi­demics in Bangladesh and have the potential to improve disease control in vulnerable areas.

## ACKNOWLEDGEMENTS

Funding for the analysis reported in this paper was provided by the NOAA Oceans and Human Health Program. The data used in this paper were collected with the support of icddr,b and its donors which provide unrestricted support to icddr,b for its operations and research. Current donors providing unrestricted support include: Australian International Development Agency (AusAID), Government of the People's Republic of Bangladesh, Canadian International Development Agency (CIDA), Swedish International Development Cooperation Agency (Sida), and the Department for International Development (DFID), UK.
